# An experimental study of magnetic compression technique for ureterovesical anastomosis in rabbits

**DOI:** 10.1038/s41598-023-27715-z

**Published:** 2023-01-31

**Authors:** Yingfeng An, Miaomiao Zhang, Shuqin Xu, Bo Deng, Aihua Shi, Yi Lyu, Xiaopeng Yan

**Affiliations:** 1 Department of Hepatobiliary Surgery, The First Affiliated Hospital of Xi’an Jiaotong University, No. 277 West Yanta Road, Xi’an, 710061 Shaanxi China; 2 National Local Joint Engineering Research Center for Precision Surgery & Regenerative Medicine, The First Affiliated Hospital of Xi’an Jiaotong University, No. 76 West Yanta Road, Xi’an, 710061 Shaanxi China; 3 Drug Non-Clinical Evaluation Center of Guangzhou Institute of Pharmaceutical Industry, Guangzhou General Pharmaceutical Research Institute Co. Ltd., Guangzhou, 510180, Guangdong China

**Keywords:** Urinary tract, Bladder, Ureter

## Abstract

This study aimed to explore the feasibility of the magnetic compression technique (MCT) for ureterovesical anastomosis in a rabbit model with ureteral obstruction. The distal ureteral obstruction model using female New Zealand rabbits was induced by ligating the distal end of the right ureter with silk thread for four weeks. A pair of cylindrical NdFeB magnets (daughter magnet and parent magnet) with a hole in the center was used for the ureterovesical anastomosis. The daughter magnet and the parent magnet were respectively placed close to the obstruction site through the dilated proximal ureter and urethra, and then the daughter-parent magnets pair was attracted together automatically. Postoperative X-rays were taken to confirm the position of the magnets. The anastomotic stoma specimens were obtained two weeks postoperatively, and the anastomotic stoma formation was observed by the naked eye and histological staining. The operation time and the anastomotic burst pressure were measured. The ureter was significantly dilated in the fourth week after ligation, which satisfied the placement of the daughter magnet. The ureterovesical magnet placements were successfully performed in ten experimental rabbits, with an operation time of 36.5 ± 6.09 min. The parent and daughter magnets attracted each other well and were subsequently removed through the urethra two weeks postoperatively, resulting in the establishment of ureterovesical anastomosis. The anastomotic burst pressure was 147.5 ± 14.59 mmHg. Gross specimens and histological examination of the anastomotic stoma showed that the anastomotic stoma healed well. MCT is feasible and simple for ureterovesical anastomosis.

## Introduction

Ureterostenosis is a complicated disease that is usually associated with hydronephrosis or nonfunctioning kidneys, leading to severe adverse effects and a poor quality of life, especially in patients after a kidney transplantation^1–3^. Conventional treatment options, including percutaneous balloon ureteroplasty, nephroureteral stenting, and surgical ureteral reconstruction have been widely applied for the treatment of benign ureteral strictures. However, each treatment has considerable limitations. Although surgical reconstruction is the gold standard, it is also associated with greater traumas and medical costs. Balloon dilatation and stenting are superior in terms of their minimal invasion, while the poor patency performance after the balloon dilatation and the necessity of long-term placement of a double-J stent negatively influences the quality of life of patients^4,5^. Therefore, novel technologies with minimal invasion and a higher quality of life for patients are needed.

Magnamosis, based on the principle of the magnetic compression technique (MCT), is a new anastomosis method that uses the noncontact magnetic field force between magnets to achieve the anastomotic reconstruction of hollow organs. Magnamosis can be used for esophageal anastomosis^6,7^, esophagogastric anastomosis^8^, gastrointestinal anastomosis^9^, colon anastomosis^10^, and biliojejunostomy magnamosis^11^ under open or laparoscopic surgery. Due to the advantages of the simple operation and reliable anastomosis effects, it is also called “smart anastomosis.” In addition to being used for conventional anastomosis, magnamosis has been reported for reconstruction and recanalization in specific conditions, such as benign biliary strictures^12,13^, rectal stenosis after rectal cancer surgery^14,15^, and congenital or acquired esophageal stricture/atresia^16,17^. However, the application of magnamosis in urinary system anastomosis have not been reported. The purpose of this study was to explore the feasibility of MCT for ureteroscopic bladder anastomosis in rabbit models with ureteral obstruction.

## Materials and methods

### Animals

Ten 6–12 months old female New Zealand rabbits, weighing 3.5–4.0 kg, were obtained from the Experimental Animal Center, College of Medicine, Xi’an Jiaotong University. All protocols and experimental procedures of this study strictly followed the Guidelines for the Care and Use of Experimental Animals issued by the Xi’an Jiaotong University Medical Center. This study was approved by the Experimental Ethics Committee of Xi’an Jiaotong University (Permit number: XJTULAC2019-1007). In this experiment, we chose the female rabbit as the animal model to facilitate placing the magnet into the bladder through the urethra. The rabbits were acclimatized to laboratory conditions (23 °C, 50% humidity, 12 h:12 h light–dark cycle, and food and water provided ad libitum) for one week before commencing the experiments. As an exploratory study, all rabbits were included in the experimental group, without a control group. Pethidine hydrochloride (1 mg/kg) was injected intramuscularly every 12 h for analgesia for three days postoperatively. At the end of the study, all rabbits were euthanized by an intravenous overdose of sodium pentobarbital (60 mg/kg) for tissue collection.

### Magnets

**Figure 1:**
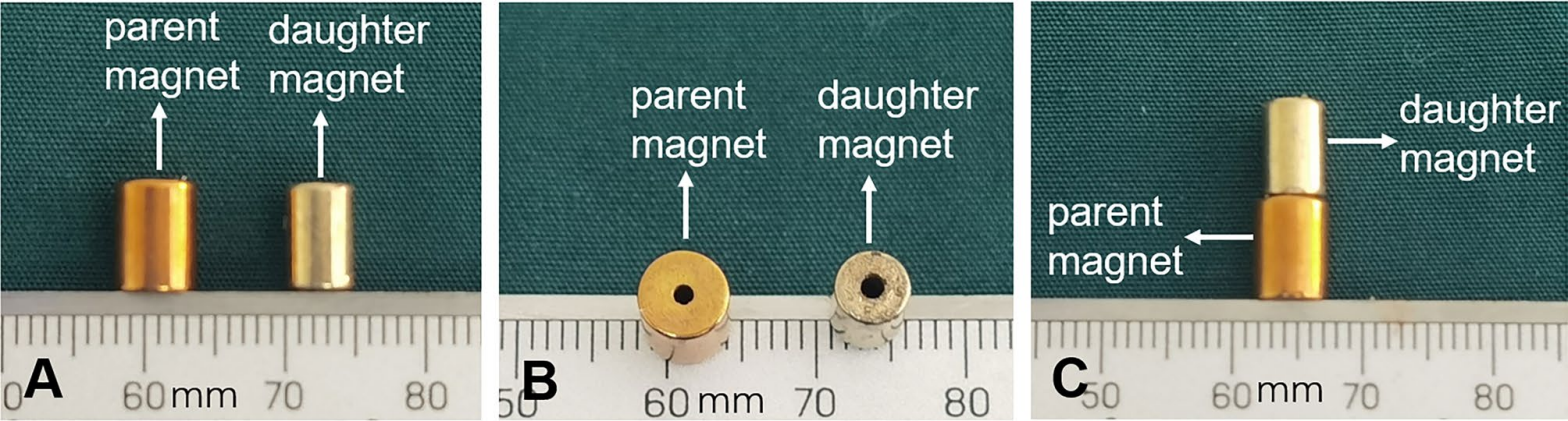
Photographs of the parent magnet and the daughter magnet. (**A**) Side view of the magnets. (**B**) Bottom view of the magnets. (**C**) Side view of the two magnets attracted together.

The magnets used for ureterovesical anastomosis consisted of a daughter magnet and a parent magnet made of neodymium-iron-boron (N45) and coated on the surface with titanium nitride. The daughter magnet was a cylinder (diameter: 5 mm; length: 8 mm; center hole diameter: 1 mm) with axial saturation magnetization, the maximal magnetic field intensity at the top and bottom surfaces is 360 mT. The parent magnet was also a small cylinder (diameter: 6 mm; length: 9 mm; center hole diameter: 1 mm) with axial saturation magnetization, the maximal magnetic field intensity at the top and bottom surfaces is 430 mT (Fig. 1). The magnetic force of the daughter magnet and the parent magnet at a spacing of 2 mm is 6.4 N. The weights of the parent magnet and daughter magnet were 1.050 g and 0.979 g, respectively. The parent and daughter magnet were manufactured by the Northwest Institute for Nonferrous Metal Research (Xi’an, Shaanxi Province, China).

### Animal model of ureteral obstruction

**Figure 2:**
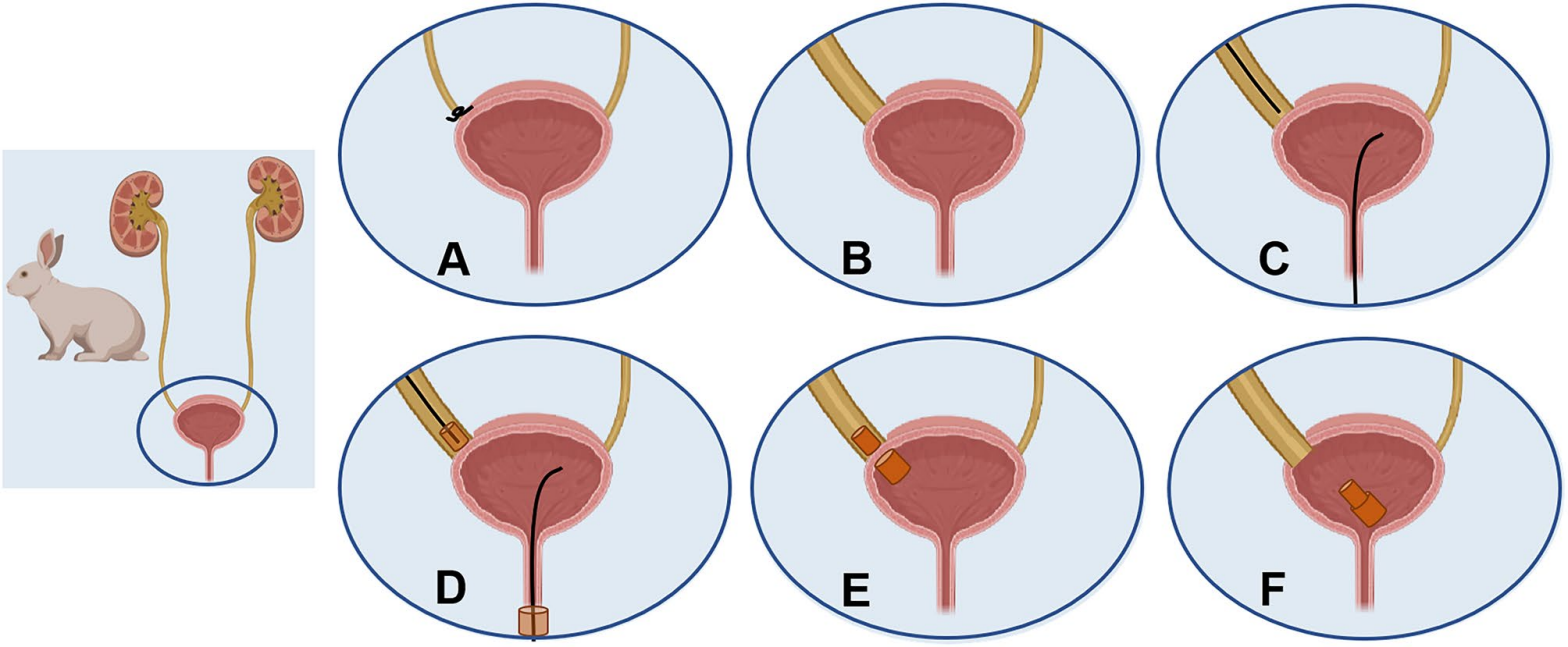
Schematic illustration of the surgical procedure. (**A**) Ligation of the right ureter. (**B**) Significant dilatation of the right ureter. (**C**) Insertion of the guide wire to the bladder and right ureter, respectively. (**D**) The daughter and parent magnets were placed along the guide wire into the right distal ureter and bladder, respectively. (**E**) The parent and daughter magnets are attracted together. (**F**) The anastomosis is established.

A midline incision of approximately 5 cm was made in the lower abdomen of the experimental rabbits. The distal end of the right ureter was dissected out and then ligated with 4–0 silk thread (Fig. 2A). According to the preliminary experimental results, at four weeks after the distal ureteral ligation, the involved ureter was able to dilate to more than 5 mm. (Fig. 2B), which satisfied the placement of the daughter magnet.

### Surgical procedures and postoperative care

Four weeks after distal ureteral ligation, the rabbits were anesthetized by an intravenous injection of 3% pentobarbital sodium (1 mL/kg) via the auricular vein according to their weight. While under anesthesia, each animal was placed in a supine position on an operating table, and the limbs were immobilized. After routine shaving and disinfection, a median incision in the lower abdomen of about 10 cm was performed. The abdominal cavity was explored to expose the affected right ureter. The upper segment of the dilated ureter was punctured with a 22G puncture needle, and then the contrast agent was injected to visualize the right ureter. Transurethral insertion of the interventional guide wire (0.035 in; TERUMO, Japan) into the bladder was performed. The upper segment of the right ureter was longitudinally incised with a length of about 5 mm, and another interventional guide wire was inserted into the proximal part of the ureteral obstruction (Fig. 2C). Pay attention to the polarity of the magnet to ensure that the daughter and parent magnets can attract each other after placement (if the polarity of the magnet is not suitable, the daughter and parent magnets will repel each other after being placed). The magnets were loaded onto the guide wires, ensuring that their polarities were aligned so as to attract after placement. The daughter magnet and the parent magnet were introduced close to the obstruction site along the guide wire through the ureter and the urethra, and then placed above the ureteral obstruction and in the bladder, respectively (Fig. 2D). At this time, the daughter-parent magnets pair could be automatically aligned and attracted (Fig. 2E). The guide wire was removed, and the parent and daughter magnets were placed in the ureter and bladder. At the same time, a 2 mm plastic tube was indwelling and fixed in the right ureter through the incision to drain the urine outside the body. The rabbits were housed individually in cages and received analgesia and anti-infective treatment for three continuous days postoperatively. X-ray examination was performed regularly after the daughter and parent magnets were placed. When the daughter and parent magnets entered the bladder, indicated the anastomosis was established (Fig. 2F). Then a catheter with a small magnet fixed at the head end was inserted into the bladder through the urethra, the small magnet attracted the daughter and the parent magnets. After pulling out the catheter, the daughter and parent magnets could be removed through the urethra. Two weeks after the magnet was removed, the animals were sacrificed, and the gross specimens of anastomosis were obtained for gross and histological observation.

### Experimental parameters

(1) *Operation time* The operation time was defined as from the opening of the abdominal cavity to the closing of the abdominal cavity with sutures. This time specifically refers to the time of magnetic ureterovesical anastomosis, while the operation time of preparation of the distal ureteral obstruction model was not calculated. (2) *Bursting pressure* The distal end of the left ureter and the proximal end of the right ureter were ligated with silk thread, and then a catheter was inserted into the bladder through the urethra and firmly fixed. The entire specimen was completely immersed in 0.9% sodium chloride solution, and air was injected through the catheter. The pressure at which bubbles overflowed from the ureterovesical anastomosis was recorded as the anastomotic burst pressure.

### Gross and histological analyses

Two weeks after the daughter and parent magnets were removed transurethral, the rabbits were euthanized by an overdose of anesthesia, and a laparotomy was performed to obtain the urethra, bladder, and right ureter. The gross specimens of the anastomosis were observed from the ureteral side and the bladder side, respectively. The anastomotic stoma was longitudinally dissected to observe the mucosal healing of the anastomotic stomal surface. The cystostomy specimens were soaked in 10% formalin overnight. After fixation, the specimens were embedded in paraffin, and 4-mm thick sections were cut from the anastomotic site. The sections were stained with hematoxylin and eosin (HE) and Masson’s trichrome staining, and then observed under a bright-field microscope.

### Statistical analysis

All data were processed using SPSS statistical 20.0 software for Windows (IBM, Chicago, IL, USA). The measurement data were reported as the mean ± standard deviation.

## Results

### Ureteral obstruction model

**Figure 3:**
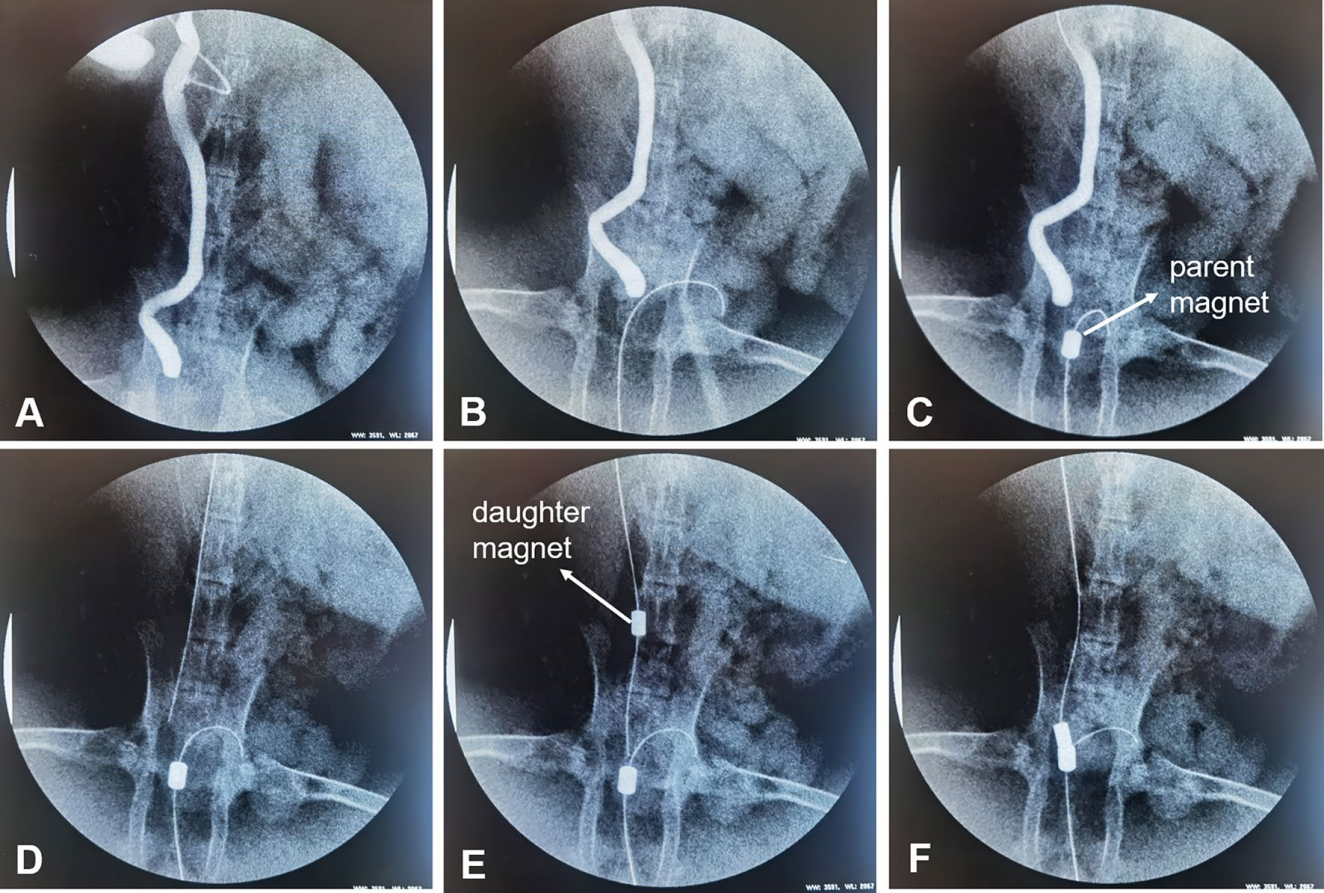
Representative intraoperative images. (**A**) Right ureterography showed distal ureteral obstruction. (**B**) The guide wire is passed through the urethra into the bladder. (**C**) The parent magnet is inserted into the bladder along the guide wire. (**D**) Guide wire into the right ureter. (**E**) The daughter magnet is inserted into the right ureter along the guide wire. (**F**) The parent and daughter magnets are attracted together.

The ureteral obstruction model was successfully induced in all ten rabbits. The right ureter was dilated to more than 5 mm after 4 weeks of ligation at the distal end of the right ureter, which satisfied the requirement of the magnamosis (Fig. 3A).

### Ureterovesical magnamosis and the procedural parameters

**Figure 4:**
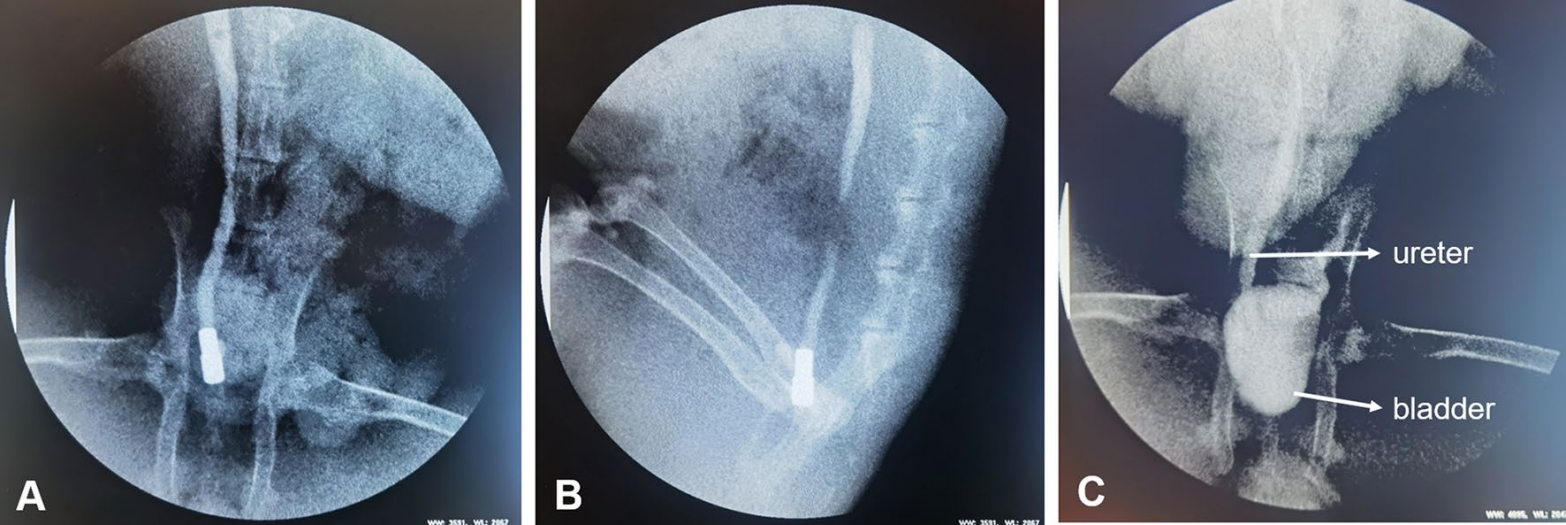
Confirmation of the position of the two magnets by ureterography. (**A**) Magnet location is shown on an abdominal radiograph; (**B**) Magnet location is shown on a lateral abdominal radiograph; (**C**) Right ureterography showing anastomotic patency.

All ten rabbits completely underwent the ureterovesical magnamosis procedure, with a success rate of 100%. Under the guidance of the interventional guide wire, the parent magnet and the daughter magnet were placed at the bladder and the distal end of the ureter, respectively (Fig. 3B–E). The daughter-parent magnets pair was automatically attracted together (Fig. 3F), and then the guide wire was removed. The entire operation time was 36.5 ± 6.09 min (range: 27–45 min). Immediate postoperative X-rays showed that the parent and daughter magnets were well attracted and precisely placed (Fig. 4A,B).

### Survival rate and postoperative complications

All rabbits survived, with a survival rate of 100% (10/10). No postoperative bleeding or infections occurred in the experimental animals. The ureterovesical anastomotic formation time was 7–10 days. Ureterography was performed and showed that the ureterovesical anastomosis was well established after the removal of the parent and daughter magnets (Fig. 4C), without complications such as anastomotic leakage or anastomotic stenosis.

### Bursting pressure

The mean burst pressure of the ureterovesical anastomosis was 147.5 ± 14.59 mmHg (range 124–165 mmHg).

### Gross and histological appearance of the anastomosis

**Figure 5:**
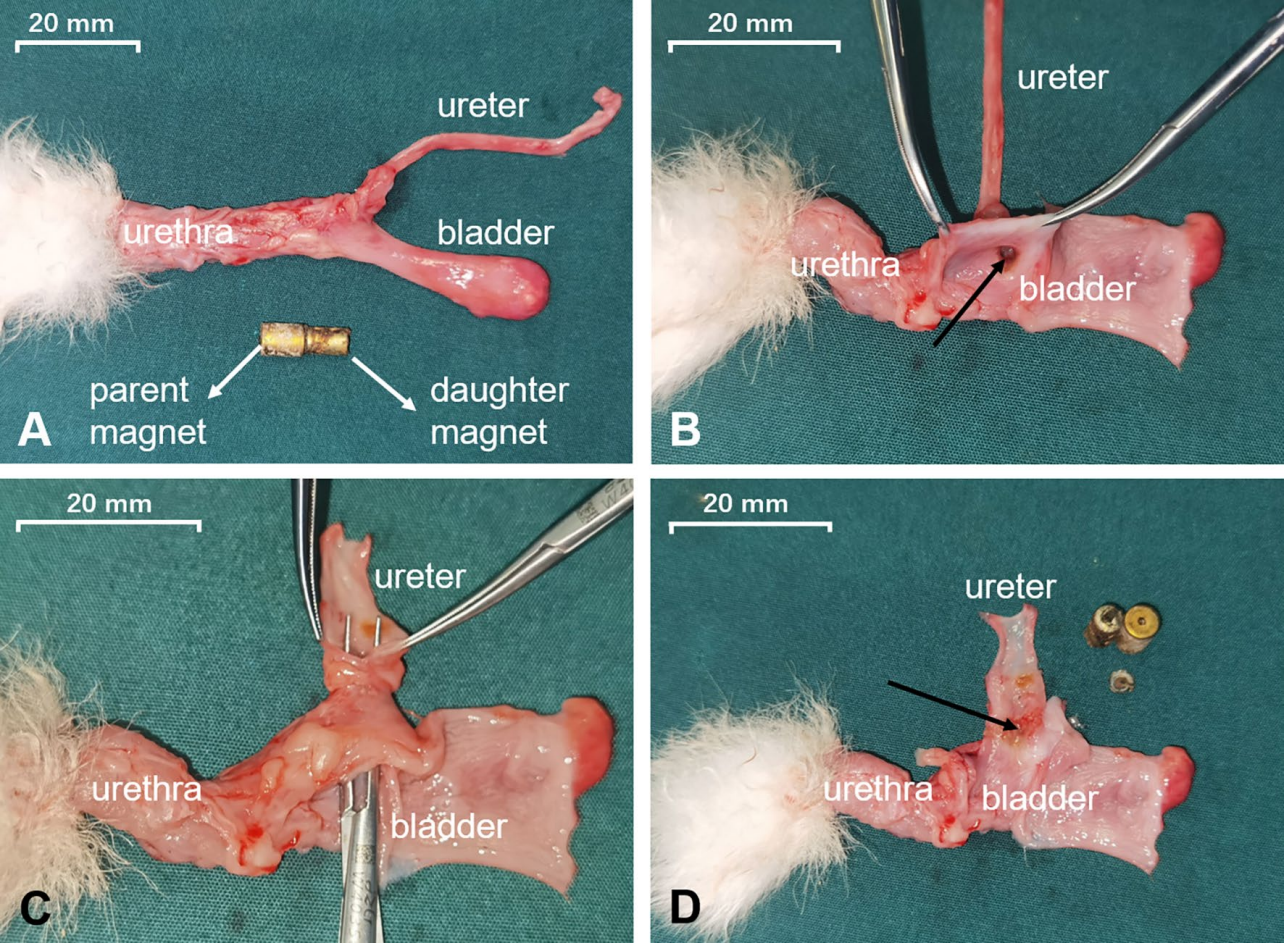
Gross specimens of ureterovesical anastomosis. (**A**) General view of the ureterovesical anastomosis. (**B**) Anastomosis on the side of the ureter. (**C**) Anastomosis on the side of the bladder. (**D**) Section view of the ureterovesical anastomosis.

**Figure 6:**
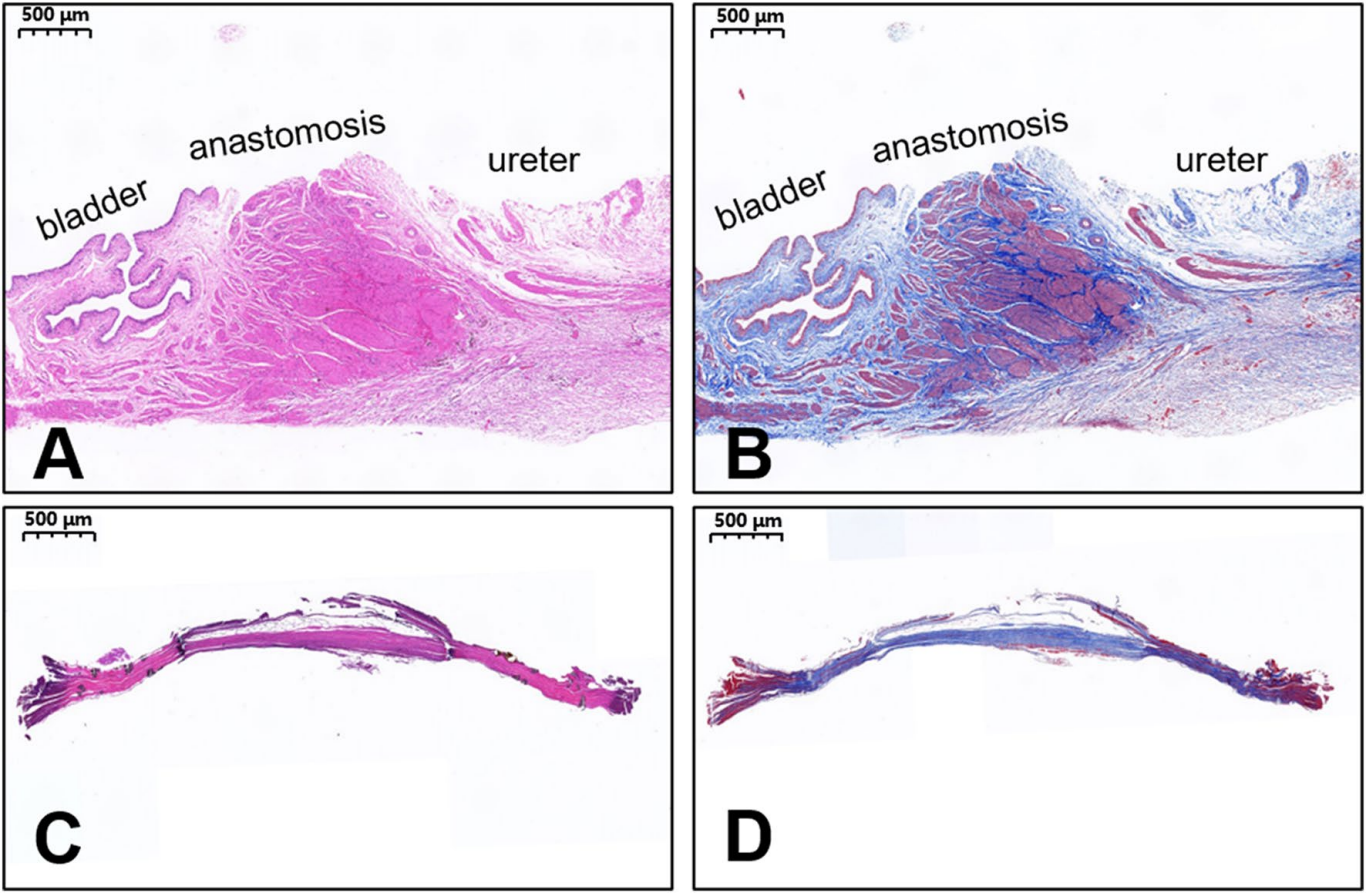
Histological images of the ureterovesical anastomosis. (**A**) HE staining, × 20 magnification; (**B**) Masson’s trichrome staining, × 20 magnification. Histological images of the necrotic tissue between the parent and daughter magnets. (**C**) HE staining, × 20 magnification; (**D**) Masson’s trichrome staining, × 20 magnification.

The gross specimen showed good patency of the ureterovesical anastomosis (Fig. 5A–D). We measured the anastomosis in gross specimens, which were approximately 4 mm in diameter. HE and Masson’s staining demonstrated the continuity of the anastomotic mucosa (Fig. 6A,B). In addition, necrotic tissue was seen between the parent and daughter magnets (Fig. 6C,D).

## Discussion

Ureteral stricture or occlusion can be caused by many benign or malignant causes, such as a history of abdominal or pelvic surgery, ureteral injury due to an endoscopic operation or ureteral calculi, retroperitoneal fibrosis, kidney transplantation, and malignancy, etc. Although conventional open ureteral reconstruction has a definite curative effect, more and more new technologies have been explored in the pursuit of minimal invasion. The current mainstream methods for ureteral stricture include laparoscopic or robotic ureteral reconstruction as well as endoscopic balloon dilation or stenting. Laparoscopic or robotic repair has achieved a comparable safety, feasibility, and success rate to open surgery, with a shorter operative time, less estimated blood loss, and significantly reduced invasion^18,19^. Recently, Yang^20^ et al. have reported that robotic ureteroplasty using lingual mucosal grafts was a feasible and safe technique for managing long, complex ureteral strictures, even in secondary ureteral reconstruction. However, the widespread implementation of laparoscopic or robotic ureteral reconstruction in general medical institutions is challenging in terms of technology, medical devices, and medical costs. Endoscopic treatment is a safe and minimally invasive alternative to surgery, but the long-term patency is still less than satisfactory. Additionally, van Son et al.^21^ have demonstrated that the patency rate of endoscopic treatment was only 27% at 60 months, which is significantly inferior to that of open surgery (69%). Moreover, Hu et al.^22^ also found that 97 of 321 (30.2%) patients undergoing balloon dilation developed restricture, with a median follow-up time of 590 days, and further revealed that the restricture of the ureter was associated with the stricture nature, urinary nitrite, chronic kidney disease, stent retention time, and balloon size. Endoscopic treatments are still not recommended for multiple, long, and recurrent ureteral strictures. Therefore, there is still a need to explore new technologies with broader indications and minimal invasion.

In the past decade, MCT has gradually been found to have potential value for the anastomosis of hollow organs. The earliest research on the magnetic compression anastomosis technique was reported in 1978, which first proved the feasibility and safety of MCT in vascular anastomosis with animal experiments^23^. However, MCT has experienced more than 20 years of long-term exploration before finally realizing the clinical value of vascular anastomosis^24^. Fortunately, during this long process of research and exploration, the potential applications of MCT have been widely described in research on the digestive system. The first human trial of magnetic compression anastomosis was reported in 2017, which showed that magnamosis was feasible and safe for intestinal anastomosis^25^. MCT was successfully used to create an anastomosis for a biliojejunostomy in 41 patients, and only 2 patients developed an anastomotic stricture with a median follow-up time of 547.5 days^11^. Furthermore, applications of MCT have not only been in the field of gastrointestinal anastomosis but also in the biliary system. The combination of MCT and endoscopic technology in the treatment of severe biliary stricture/atresia after liver transplantation has been shown to be the most disruptive innovative technology with clinical value^26^. The anatomical and pathophysiological characteristics of a ureteral stricture after kidney transplantation are very similar to those of a biliary stricture after liver transplantation. However, there are no research reports on the treatment of ureteral obstruction by MCT.

In previous studies, we have completed a large number of experimental studies and clinical applications of magnamosis in the digestive system^27,28^. Based on the satisfactory results conducted from these studies, we first proposed using MCT for ureterovesical anastomosis in the treatment of ureteral stricture/atresia. To induce the pathophysiological characteristics of patients with clinical ureteral obstruction as much as possible, we established an animal model of ureteral obstruction by ligating the distal end of the ureter with silk thread for 4 weeks. The experimental results showed that our approach was effective in inducing the clinical model of distal ureteral obstruction disease and meeting the requirements for the subsequent placement of magnets. In clinical practice, a nephrostomy is often performed in patients with ureteral obstruction when the obstruction cannot be relieved in a short term. Therefore, we proposed a dual-path magnet placement scheme to utilize the gradually expanded ostomy channel as the implantation path of the daughter magnet. In addition, the urethra could serve as a natural channel for the placement of the parent magnet. However, it was difficult to complete a nephrostomy with rabbit models in this experiment, so we inserted a guide wire and daughter magnet through the upper ureter approach in the laparotomy. The results demonstrated in this work provide a new perspective on the implication of MCT in the urinary system. To the best of our knowledge, this is the first animal experiment on MCT in ureteral stricture disease. Our results suggest that MCT may be a promising alternative to surgical and endoscopic options for ureteral restrictions. A highlight of this study lies in the unique cylindrical design of the parent and daughter magnets with a central hole, which has the following advantages: (1) The central hole of the magnet can be used as the access for the guide wire during the operation, ensuring that the magnet is accurately placed in the target position under the guidance of the guide wire; (2) a cylinder is more suitable for the anatomical characteristics of lumen-like organs and facilitates the placement of a magnet; (3) compared with other shapes of magnets, the cylindrical design enables the ends of the magnet to obtain the maximal magnetic force as possible, which is more in line with the laws of magnetism.

This study also has some limitations that must be addressed. First, the ureterovesical anastomosis could not be completed under minimally invasive conditions due to the selected animal model. The anatomical characteristics of rabbits increased the difficulty of implementing the nephrostomy. If large-scale experimental animals (beagles or pigs) would be selected, it is expected that the ureterovesical anastomosis would be performed in a more minimally invasive way. As a compensation measure, we adopted the upper ureteral approach to insert the guide wire to simulate the minimally invasive procedure as much as possible. Fortunately, the experimental results did not affect the observation and analysis of the feasibility and efficiency of magnamosis for ureteral strictures in a rabbit model. Second, the long-term patency of the ureterovesical anastomosis established by MCT was uncertain. Nevertheless, we believe that in the case of complete distal ureteral obstruction and due to the lack of other effective minimally invasive options, MCT can provide an effective method to open the obstructed channel and create a valuable opportunity for further treatments, such as indwelling double J tube or ureteral stents under cystoscopy. Furthermore, whether using MCT to directly establish an anastomosis between the ureter and the bladder would increase the risks of urine reflux and retrograde infection requires further investigation. These limitations remain to be studied in subsequent works.

## Conclusions

In conclusion, MCT is a feasible and safe method for ureterovesical anastomosis in rabbits, thus providing a new avenue for ureterovesical anastomosis with the advantages of minimal invasion and an easy operation. It is expected to be applied in clinical practice after further improvement and optimization in additional human studies.

## Data Availability

The data underlying this article will be shared on reasonable request to the corresponding author.
